# The effects of *Pediococcus acidilactici* MA18/5M on growth performance, gut integrity, and immune response using *in vitro* and *in vivo* Pacific salmonid models

**DOI:** 10.3389/fimmu.2024.1306458

**Published:** 2024-03-27

**Authors:** Manuel Soto-Dávila, Luana Langlois Fiorotto, John W. Heath, John S. Lumsden, Gregor Reid, Brian Dixon

**Affiliations:** ^1^Department of Biology, University of Waterloo, Waterloo, ON, Canada; ^2^Canadian Centre for Human Microbiome and Probiotics Research, Lawson Health Research Institute, London, ON, Canada; ^3^Departments of Microbiology and Immunology, and Surgery, The University of Western Ontario, London, ON, Canada; ^4^Yellow Island Aquaculture, Heriot Bay, BC, Canada; ^5^Department of Pathobiology, University of Guelph, Guelph, ON, Canada; ^6^Department of Surgery, The University of Western Ontario, St. Joseph’s Health Care London, London, ON, Canada

**Keywords:** RTgutGC, Chinook salmon, *Pediococcus acidilactici* MA 18/5M, immune response, gut barrier function, tight junctions, histopathological analysis, Transwell

## Abstract

Microbial management is central to aquaculture’s efficiency. *Pediococcus acidilactici* MA18/5M has shown promising results promoting growth, modulation of the immune response, and disease resistance in many fishes. However, the mechanisms through which this strain confers health benefits in fish are poorly understood, particularly in Pacific salmonid models. Briefly, the aims of this study were to i) assess the protective effects of *P. acidilactici* MA18/5M by examining gut barrier function and the expression of tight junction (TJ) and immune genes *in vitro* and *in vivo*, and ii) to determine the protective effects of this strain against a common saltwater pathogen, *Vibrio anguillarum* J382. An *in vitro* model of the salmonid gut was employed utilizing the cell line RTgutGC. Barrier formation and integrity assessed by TEER measurements in RTgutGC, showed a significant decrease in resistance in cells exposed only to *V. anguillarum* J382 for 24 h, but pre-treatment with *P. acidilactici* MA18/5M for 48 h mitigated these effects. While *P. acidilactici* MA18/5M did not significantly upregulate tight junction and immune molecules, pre-treatment with this strain protected against pathogen-induced insults to the gut barrier. In particular, the expression of *ocldn* was significantly induced by *V. anguillarum* J382, suggesting that this molecule might play a role in the host response against this pathogen. To corroborate these observations in live fish, the effects of *P. acidilactici* MA18/5M was evaluated in Chinook salmon reared in real aquaculture conditions. Supplementation with *P. acidilactici* MA18/5M had no effect on Chinook salmon growth parameters after 10 weeks. Interestingly, histopathological results did not show alterations associated with *P. acidilactici* MA18/5M supplementation, indicating that this strain is safe to be used in the industry. Finally, the expression pattern of transcripts encoding TJ and immune genes in all the treatments suggest that variation in expression is more likely to be due to developmental processes rather than *P. acidilactici* MA18/5M supplementation. Overall, our results showed that *P. acidilactici* MA18/5M is a safe strain for use in fish production, however, to assess the effects on growth and immune response previously observed in other salmonid species, an assessment in adult fish is needed.

## 1 Introduction

In finfish aquaculture, the incorporation of healthy functional constituents into aquafeeds has become a potential tool to improve fish growth, stress tolerance, and disease resistance (1–3). Functional food components have been demonstrated to increase the respiratory burst activity, cytokine activity, complement system, phagocytosis, among others (4–7). To date, several immunostimulants and functional feed ingredients have been tested in aquaculture species, among which prebiotics, probiotics, vitamins, and synbiotics are notable (6, 8–11). Probiotic strains have been broadly studied in terrestrial and aquatic species and have shown promising results for the pork, poultry, and aquaculture industry (7, 12–15). This non‐polluting and efficient biological alternative to antibiotics is commercially available often at reasonable prices, making them a potential alternative for large-scale production (7–9, 14).

Probiotics are defined as “live microorganisms that, when administered in adequate amounts, confer a health benefit on the host” (16). In fish, some probiotic strains have been shown to improve fish growth and feed conversion rates, modulate the gastrointestinal microbial community, prevent bacterial diseases, and contribute to the digestive and nutritional processes of the host (15, 17–19). The fish gut constitutes the largest interface for host-microbe interactions and harbors the most abundant and diverse microbial community that can modulate overall host physiology (12). Therefore, modulation of host health at this interface may prove advantageous in aquaculture systems where optimal fish health is directly associated with profits in this highly margin-sensitive industry.

Recent reports have pointed at the potential of using *Pediococcus acidilactici* MA18/5M in terrestrial livestock as well as marine and freshwater fish, particularly salmonid species such as Atlantic salmon (20). *P*. *acidilactici* MA18/5M is a homofermentative Gram-positive cocci and is a member of the lactic acid bacteria (LAB) group. This strain has shown promising results promoting growth performance, modulation of both innate and adaptive immunity, improved survival rates, and resistance to infection (12, 17–20). To date, available research has demonstrated that *P. acidilactici* increases growth, blood leucocyte levels, immunoglobulin (Ig) levels, serum lysozyme activity, and transcripts encoding *il1b*, *il8*, *il10*, and *tnfa*, among others, in salmonids species utilized in aquaculture such as rainbow trout (*Oncorhynchus mykiss*) (21–23) and Atlantic salmon (*Salmo salar*) (1, 24–26).

The European Union has approved the use of this probiotic for aquaculture applications. Still, data supporting the use in North American species has yet to be recognized by the local regulatory agencies (18, 21). Interestingly, the Canadian Food Inspection Agency (CFIA) recently conducted consultations to evaluate the use of dehydrated *P. acidilactici* in gut modifier products for livestock species, including fish (27). In salmonids, the administration of this probiotic strain has been associated with a robust nonspecific immune response (both at the mucosal interface and at a systemic level), improving growth performance, modifying microbiota composition, and increasing disease resistance (20, 22, 23). The applicability of this strain for health enhancement of Pacific salmonid species has yet to be understood.

Salmonids constitute the most economically important family of finfish. In particular, Chinook salmon (*Oncorhynchus tshawytscha*) is the largest species of this family and presents potential economic and environmental advantages compared to farming of its Atlantic counterpart on the Pacific Northwest (28–31). However, Chinook salmon farming is hampered by the risk of escapees diluting the genetic diversity of wild populations and their poor tolerance to commercial production conditions (25, 26). Sterile triploid salmon effectively circumvents escapee issues, however these fish exhibit 10-30% higher mortality rate due to infectious disease compared to diploid fish (Data provided by Yellow Island Aquaculture Ltd.).

The fish gut is thought to be a primary site for pathogen attachment, proliferation, and entry into the bloodstream (32, 33). It serves as the primary site for digestion and nutrient absorption, immune modulation, osmoregulation, and acts as a barrier against pathobionts in the gut (33, 34). Notably, *Vibrio anguillarum* is a common aquatic pathogen that afflicts farmed fish worldwide, causing substantial financial losses to the industry (35). Therefore, investigating mechanisms that can prevent pathogen expansion, bolster gut barrier function and integrity will potentially prevent infections and sustainably improve productivity. There is currently no evidence for the effect of probiotic strain MA18/5M or other beneficial microbes in Chinook salmon.

Recent reports on a salmonid intestinal epithelial cell line derived from rainbow trout (*O. mykiss*), RTgutGC, have established the usefulness of this cell line as a model for functional studies on fish feed development based on gut barrier function and immune competence, as well as host-microbe interactions with viruses (36–40). Furthermore, by employing semipermeable membrane supports (Transwell^®^), it is possible to recapitulate the intestinal environment *in vitro* and conduct studies on the permeability and integrity of the cell monolayer (41).

The objectives of the present study are to i) investigate the effect of *P*. *acidilactici* MA18/5M supplementation on gut barrier function and the expression of tight junction and immune molecules, ii) determine the extent to which *V. anguillarum* disrupts barrier function, iii) assess whether *P. acidilactici* MA18/5M can protect monolayer integrity against pathogen-induced insults to the barrier, and iv) examine the effects of *P. acidilactici* MA18/5M on several physiological parameters of juvenile Chinook salmon.

## 2 Materials and methods

### 2.1 In vitro P. acidilactici MA18/5M trials

#### 2.1.1 Tissue culture maintenance

The rainbow trout intestinal epithelial cell line RTgutGC was cultured in Leibovitz’s 15 media (HyClone, Cytiva), supplemented with 10% heat-inactivated fetal bovine serum (HI-FBS; Life Technologies), and incubated in plates or T75 flasks (Corning, Millipore Sigma) sealed with Parafilm (Bemis) at 22°C and atmospheric conditions. The medium was replaced every 3-4 days and cells were passaged when ≥80% confluent in a 1:2 to 1:4 sub-cultivation ratio, depending on the downstream experimental application. Cells were washed with 4 mL sterile PBS at room temperature, and residual buffer was aspirated with a glass Pasteur pipette (Fisher Scientific) connected to a vacuum line. Four mL of trypsin (0.05% w/v; Thermo Fisher Scientific) was then added and incubated for 10 min at room temperature on a flask vortex to facilitate detachment.

Cells were monitored periodically using a Nikon inverted microscope to ensure detachment from the plastic. Upon detachment, 8 mL of complete culture medium (L-15 + 10% FBS) was added to quench the trypsin protease activity. The suspension was then vigorously pipetted to break up clumps of cells, before the transfer of the cells to a 15 mL sterile conical tube from which a sample was taken for viable counting using a trypan blue (0.04%; Thermo Fisher Scientific) exclusion assay and the automated Countess cell counter (Invitrogen) prior to seeding into the cell culture dishes. The medium renewal was performed 24-48 h following trypsinization and seeding into new culture dishes to remove residual trypsin.

#### 2.1.2 Bacterial strains and culture conditions

To determine the probiotic effect on RTgutGC, *P. acidilactici* MA18/5M (BioPower^®^ PA, registration number 982989) was utilized. This strain was generously provided as a lyophilized powder by Lallemand Animal Nutrition Incorporated. *P. acidilactici* MA18/5M was routinely cultured anaerobically at 37°C in Mann, Rogosa, and Sharpe (MRS) medium (BD Difco) for each of the trials.

For RTgutGC cell stimulation trials, the Gram-negative pathogen *V. anguillarum* J382 (serotype O1) (42) isolated from winter Steelhead trout was obtained from Little Campbell River (British Columbia, Canada) and utilized for cell stimulation. Briefly, a single colony of *V. anguillarum* was grown in 2.5 mL of trypticase soy broth 2% sodium chloride (TSB 2% NaCl; Multicell Wisent, Quebec, Canada) at 20°C in a 16 mm diameter glass tube and placed in a shaker for 24 h at 200 rpm. After growth, 150 μL of the overnight culture were added to 150 mL of TSB 2% NaCl media using a 250 mL flask and incubated for 24 h at 20°C with aeration (200 rpm). After the overnight culture, the bacterial inoculum was centrifuged at 6,000 rpm at room temperature for 10 min. The pellet was washed thrice with PBS and centrifuged at 6,000 rpm at room temperature for 10 min, and finally resuspended in 25 mL of PBS (~8.6 × 10^8^ CFU mL^−1^). The concentrated bacterial inoculum was serial diluted and quantified by plating onto TSA 2% NaCl for 48 h. Heat-killed *V. anguillarum* was prepared by transferring 1 mL of this inoculum to a 1.7 mL tube, which was then centrifuged at 10,000 rpm for 8 min at room temperature. The supernatant was discarded, and the bacterial pellet was resuspended in 1 mL of sterile PBS and incubated at 100°C for 30 min. Then, 100 μL of the heat-killed suspension was plated in TSA + 2% NaCl in triplicates to ensure sterility.

#### 2.1.3 Coculture experiments

**Figure 1:**
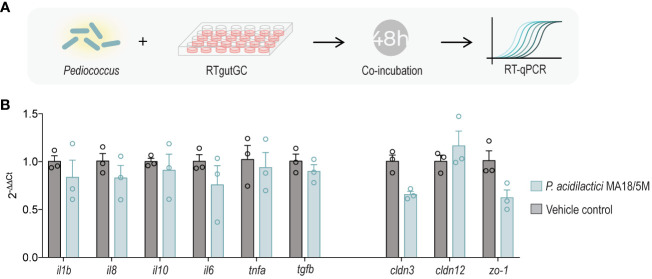
Coincubation of *P. acidilactici* MA18/5M with RTgutGC to evaluate expression of immune and barrier genes **(A)**. Relative gene expression of interleukin 1 beta (*il1b*), interleukin 8 (*il8*), interleukin 10 (*il10*), interleukin 6 (*il6*), tumor necrosis factor alpha (*tnfa*), transforming growth factor beta (*tgfb*), claudin 3 (*cldn3*), claudin 12 (*cldn12*), and zonula occludens 1 (*zo-1*) in RTgutGC unstimulated (control) or inoculated with *P. acidilactici* MA18/5M for 48 h **(B)**. All data are expressed as mean values ± S.E.M (n=3). Differences were not statistically significant (Kruskal-Wallis non-parametric test).

**Figure 2:**
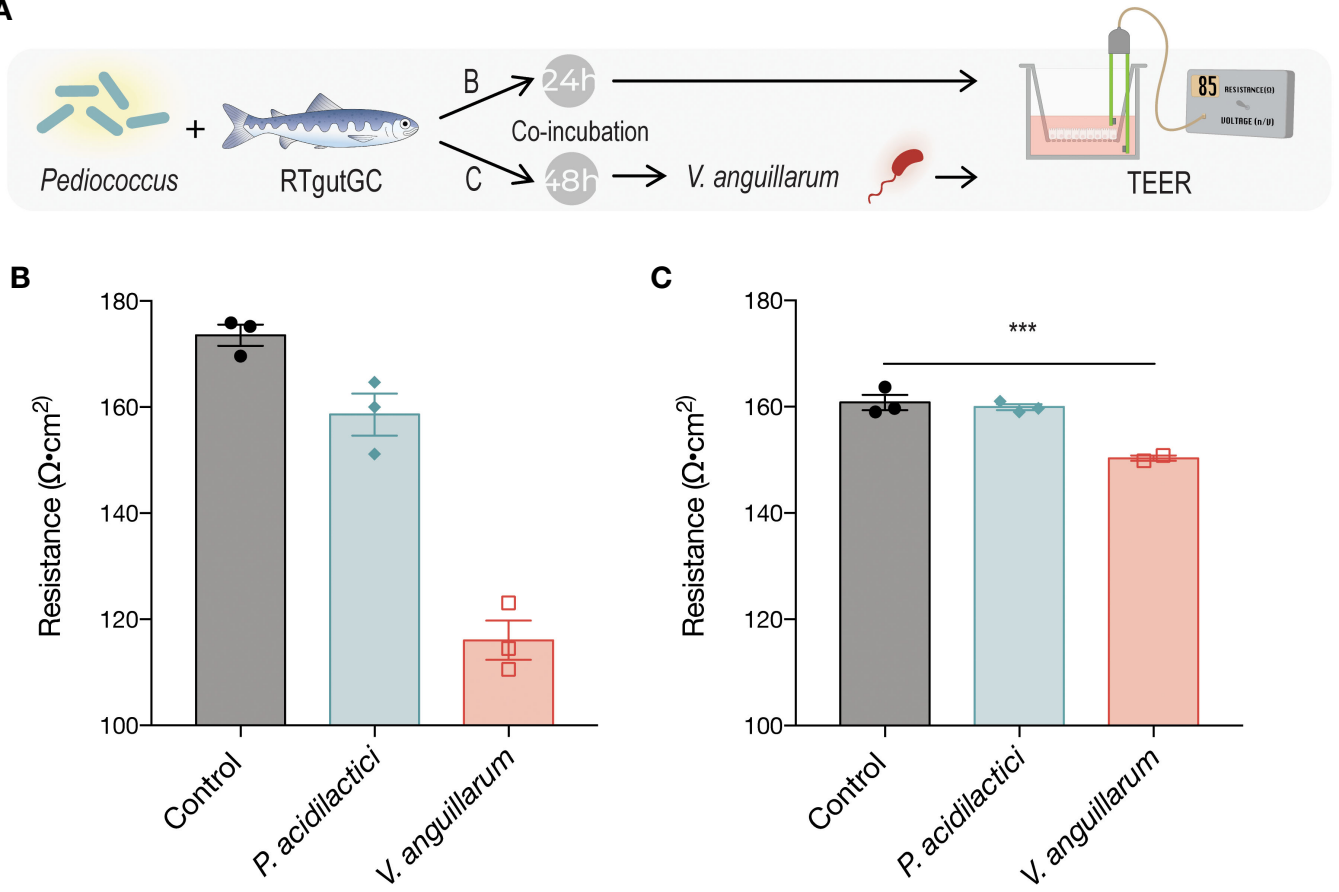
Transepithelial electrical resistance (TEER) in response to exposure to *P. acidilactici* MA18/5M or *V. anguillarum* J382 **(A)**. RTgutGC cells were seeded on Transwell semipermeable transmembrane supports and *P. acidilactici* MA18/5M was added to the apical compartment for either 24 h **(B)** or 48 h **(C)**. The control group was not exposed to bacteria at any time, and the *V. anguillarum* J382 group was incubated only with the bacterium for the latter 24 h of the experiment **(B, C)**. TEER was determined based on the resistance given by the monolayer per area, normalized to the blank measurement. Bars indicate statistically significantly differences among treatments (*P < 0.05, ** P < 0.01, *** P < 0.001).

**Figure 3:**
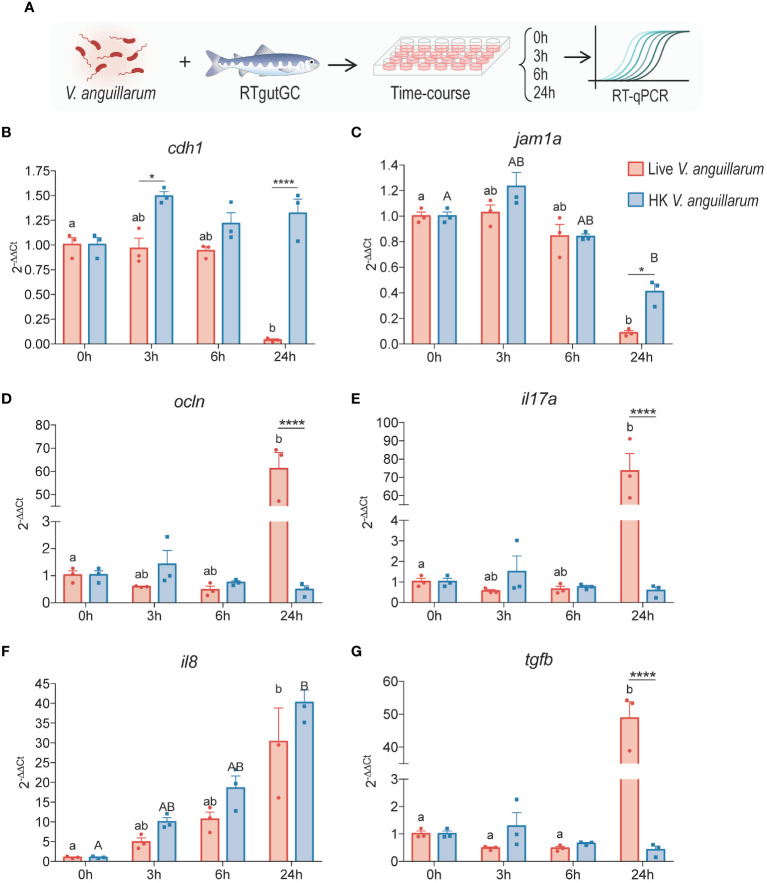
Time-course analysis of salmonid intestinal cells to live or heat-killed *V. anguillarum*. RTgutGC cells were exposed to either live or HK bacteria (2:1 MOB at the time of inoculation) **(A)** and gene expression of **(B)** e-cadherin (*cdh1*), **(C)** junctional adhesion molecule 1 alpha (*jam1a*), **(D)** occludin (*ocln*), **(E)** interleukin 17 (*il17a*), **(F)** interleukin 8 (*il8*), and **(G)** transforming growth factor β (*tgfb*) were measured using RT-qPCR. All data are expressed as mean values ± S.E.M (n=3). Bars represent significant differences between treatments at the same time-points. Different letters (Live bacteria: lower cases; HK bacteria: Upper cases) represent significant differences in each treatments at different times (*P < 0.05, ** P < 0.01, *** P < 0.001).

**Figure 4:**
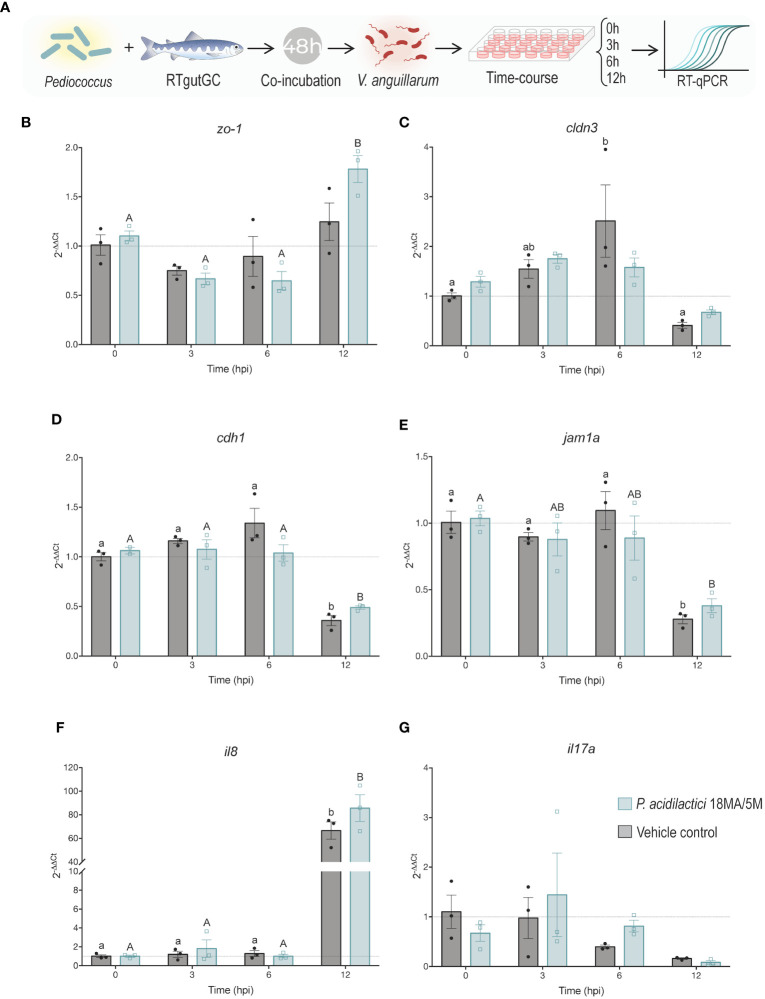
Effect of pre-treatment with *P. acidilactici* MA18/5M followed by *V. anguillarum* inoculation on the expression of key tight junction and immune molecules. **(A)** RTgutGC intestinal epithelial cells were incubated with *P. acidilactici* MA18/5M for 48 h (MOB 1:100; ~7.5 x 10^8^ CFU/mL), then infected with *V. anguillarum* (2:1 MOB at the time of inoculation) and samples were collected at 0, 3, 6, and 12 h post-infection. The control group was not pretreated with *P. acidilactici* MA18/5M at any time and was only exposed to *V. anguillarum* for the latter 12 h of the experiment. Gene expression of **(B)** zonula occludens-1 (*zo-1*), **(C)** claudin 3 (*cldn3*), **(D)** e-cadherin (*cdh1*), **(E)** junctional adhesion molecule 1 alpha (*jam1a*), **(F)** interleukin 8 (*il8*), and **(G)** interleukin 17 (*il17a*) were assessed using RT-qPCR. All data are expressed as mean values ± S.E.M (n=3). Different letters (control: lower cases; *P. acidilactici* MA18/5M: Upper cases) represent significant differences in each treatments at different times (P < 0.05).

RTgutGC cells were cultured in 6- or 12-well plates (BD Falcon) for at least 3 weeks prior to the experiments to ensure that the cells established the brush border membrane and tight junction complexes. Frozen stocks of *P. acidilactici* MA18/5M and *V. anguillarum* J382 were streaked onto agar plates of the appropriate medium and incubated for 24 h. Single colonies were then re-streaked and incubated for another 24 h. Fresh single colonies were used to inoculate 3 mL of the appropriate growth medium and cultures were incubated for 48 h. Assuming that the concentration per area of cells at confluency is approximately 1.3 x 10^5^ cells/cm^2^, the *P. acidilactici* MA18/5M was diluted to a final multiplicity of bacteria (MOB) of 1:100 gut cells to bacteria, while *V. anguillarum* was diluted to a final concentration of 2:1 MOB. Heat-killed *V. anguillarum* was diluted in like manner. The bacterial suspensions were mixed in the cell culture growth medium, and the spent cell culture medium was aspirated using a sterile glass Pasteur pipette connected to a vacuum line. The bacteria were then added to the RTgutGC cells and incubated for various durations (**Figures 1**–**4**). Cells were then harvested at specific time-points by aspirating the culture medium and adding 1 or 0.5 mL (for 6- or 12-well plates, respectively) of TRIzol reagent (Invitrogen), and removed by vigorously pipetting the RTgutGC cell lysate, which was then transferred to a 1.7 mL tube and stored at 4°C until further processing.

#### 2.1.4 RTgutGC RNA extraction and cDNA synthesis

Total RNA was isolated from RTgutGC cell lysates using 0.3 volumes of chloroform per 1 volume of TRIzol (Invitrogen, 2020). After this, 0.7 volumes of 100% isopropanol per 1 volume of the sample were added, vortexed briefly, and incubated at room temperature for 5 min. Samples were then centrifuged at 16,000 rpm for 15 min at 4°C. The supernatant was discarded, and residual isopropanol was removed. Then, 1 mL of 70% ethanol in nuclease-free water (Invitrogen) was added and samples were centrifuged at 16,000 rpm for 15 min at 4°C. The residual ethanol was carefully removed, and pellets were air-dried for 15 to 20 min. The RNA was then resuspended in 30 μL of warm (56°C) nuclease-free water and DNase treated with Ambion DNase I (RNase free) (Ambion™ DNase I, Invitrogen) following the manufacturer’s instructions to degrade any residual genomic DNA. Then, DNase treated RNA samples were quantified using a NanoDrop spectrophotometer. Purified RNA samples had A260/280 ratios between 1.9 and 2.1 and A260/230 ratios between 1.9 and 2.2.

First-strand cDNA templates for qPCR were synthesized from 1 μg of the freshly isolated RNA using a High-Capacity cDNA Reverse Transcription Kit, following the manufacturer’s instructions (Applied Biosystems). Each sample was incubated at 25°C for 10 min, at 37°C for 120 min, and at 85°C for 2 min. Samples with a total volume of 40 μL per reaction were stored at -80°C until utilization.

#### 2.1.5 qPCR analysis of RTgutGC samples

All qPCR reactions were performed in a 10 μL reaction, 4.58 μL of 1:10 diluted cDNA, 0.42 μL of primers (forward and reverse primer mix; 14.4 μM), and 5 μL of Power SYBR Green 2x Master Mix. All samples were amplified and detected using the QuantStudio5 Real-Time PCR System (Thermo Fisher Scientific) and analyzed using the associated cloud-based Design and Analysis software (Thermo Fisher Scientific; version 2.5.1). The reaction conditions were 50°C for 2 min, then 95°C for 10 min, followed by 40 cycles of 95°C for 15 s, then 60°C for 1 min. The melt curve was assessed every 15 s between 60-95°C.

The primer sequences of interleukin 1 beta (*il1b*), interleukin 6 (*il6*), interleukin 8 (*il8*), interleukin 10 (*il10*), interleukin 17a (*il17a*), tumor necrosis factor alpha (*tnfa*), transforming growth factor β (*tgfb*), E-cadherin (*cdh1*), claudin 3 (*cldn3*), claudin 12 (*cldn12*), occludin (*ocln*), junctional adhesion molecule 1 alpha (*jam1a*), zonula occludens-1 (*zo-1*), used in this study are listed in **Table 1**. Gene nomenclature abbreviations were obtained from the Zebrafish Information Network database (www.zfin.org). Since the reagents, cycling conditions and samples were different from previous studies, primer efficiencies were measured. Briefly, a 7-point 1:3 dilution series starting with cDNA representing 5 ng/µL (12.5 ng per reaction) of input total RNA was generated, and efficiencies then calculated using the formula E = 10^(−1/slope)^.

**Table 1:** Primers used in this study.

Gene name	Sequence (5’-3’)	Efficiency (%)	Amplicon size (bp)	Reference
Interleukin 1 beta (*il1b*)	F: GCTGGAGAGTGCTGTGGAAGA R: TGCTTCCCTCCTGCTCGTAG	100.1	73	(43)
Interleukin 6 (*il6*)	F: GTTCTGGGTGAGGTGTCTA R: GGTGTCAACCAGGAAGTTAC		93	(44)
Interleukin 8 (*il8*)	F: ATTGAGACGGAAAGCAGACG R: CGCTGACATCCAGACAAATCT	98.2	136	(43)
Interleukin 10 (*il10*)	F: CCATCAGAGACTACTACGAGGC R: TCTGTGTTCTGTTGTTCATGGC	102.2	165	(43)
Interleukin 17a (*il17a*)	F: TGGTTGTGTGCTGTGTGTCTATGC R: TTTCCCTCTGATTCCTCTGTGGG		136	(43)
Tumor necrosis factor alpha (*tnfa*)	F: GTGATGCTGAGTCCGAAAT R: GTCTCAGTCCACAGTTTGTC	93.6	97	(45)
Myeloid differentiation factor 88 (*myd88*)	F: GACAAAGTTTGCCCTCAGTCTCT R: CCGTCAGGAACCTCAGGATACT	97.5	110	This study
Transforming growth factor β (*tgfb*)	F: AGTTGCCTTGTGATTGTGGGA R: CTCTTCAGTAGTGGTTTGTCG	92.1	191	This study
Tricellulin (*marveld2*)	F: TCCAACACAGGCTCATCTCTT R: ATGGGGTTCATGACGGACAC	99.3	83	This study
E-cadherin (*cdh1*)	F: ACTACGACGAGGAGGGAGGT R: TGGAGCGATGTCATTACGGA	96.7	107	This study
Villin 1 (*vil1*)	F: AAAGTTCAGGTGCTGTAAATCGC R: TGTGGCATGGTGCCAGATTC	105.6	148	This study
Claudin 3 (*cldn3*)	F: AGGCAACGACGCTACATCAA R: GAAACCCAAGCAATGCGTCA	100.4	112	(39)
Claudin 12 (*cldn12*)	F: ATCATCGCCTTCATCTCCGT R: TAGCAGCCAGAGTAGCCATC	91.5	161	This study
Claudin 15 (*cldn15*)	F: GGCACGTCTGAGAAACAACC R: TAGGAAGTGGCAGCCTGACT	102.8	92	This study
Occludin (*ocln*)	F: F: GACAGTGAGTTCCCCACCAT R: AGCTCTCCCTGCAGGTCCTT	103.1	101	This study
Junctional adhesion molecule 1 alpha (*jam1a*)	F: TGAGGATGGAAGTCCGCAAC R: GTACCACAGTCCGAAGCACA	101.9	98	This study
Zonula occludens-1 (*zo-1*)	F: GCTGTTCCTCCTAGACCTT R: TCACCCACATCTGACTCTAC	99.4	99	(44)
Mucin 2 (*muc2*)	F: CCAGTGTCAGTGCAAACACG R: ATGTAGCAGGGCTGGGTAGA	100.6	122	This study
^a,c^Cytochrome c oxidase subunit 6C-1 (*cx6c1*)	F: GCCTGCAATGCGAGGACTCC R: TTCCTTGGTTCTGTTACGCCGTAC	104.2	114	This study
^b,c,d^Elongation factor 1 alpha (*ef1a*)	F: CGCACAGTAACACCGAAACTAATTAAGC R: GCCTCCGCACTTGTAGATCAGATG	99.1	134	(45)
^b,c,d^Beta actin (*actb*)	F: TGGACTTTGAGCAGGAGATGG R: AGGAAGGAGGGCTGGAAGAG	90.2	139	(46)
^b,c,d^Glyceraldehyde-3-phosphate dehydrogenase (*gapdh*)	F: GCTGGAATGGGACTCACAC R: GTCAAAACCGTCTCAGTGGG	100.8	NR	(47)
^b.d^18S ribosomal RNA (*18S*)	F: CGTCGTAGTTCCGACCATAAA R: CCACCCACAGAATCGAGAAA	101.4	NR	(48)
^b,c,d^Internal transcribed spacer 2 locus (*its2*)	F: TCATCAATCGGAACCTCTGG R: AAGGAAGAGCGCACGGG	98.6	156	(49)

a Normalizers used in experimental RTgutGC qPCR analyses.

b Normalizers used in experimental Chinook salmon qPCR analyses.

c Candidate normalizer genes for in vitro trial.

d Candidate normalizer genes for in vivo trial.

NR: Not reported.

Transcripts levels of the genes of interest (*il1b*, *il6*, *il8*, *il10*, *il17a*, *tnfa*, *tgfb*, *cdh-1*, *cldn3*, *cldn12*, *ocln*, *jam1a*, and *zo-1*) were normalized to transcript levels of the endogenous control gene cytochrome c oxidase subunit 6C-1 (*cx6c1*). Levels of five candidate normalizers [*cx6c1*, elongation factor 1 alpha (*ef1a*), beta actin (*actb*), glyceraldehyde-3-phosphate dehydrogenase (*gapdh*), and internal transcribed spacer 2 locus (*its2*)] were assessed. After normalizer testing was completed, transcript levels of the genes of interest were analyzed in the individual study samples, with normalization to *cx6c1*. On each gene a no RT control was included. Gene expression was determined using the comparative 2^−ΔΔCt^ method (50).

#### 2.1.6 Transepithelial electrical resistance

To determine the change in epithelial electrical resistance given by the effect of different treatments on the cell monolayer, a TEER experiment was carried out (**Figure 2A**). Prior to seeding cells onto the Corning Transwell polyester membrane cell culture inserts (6.5 mm and 0.4 μm pore size), baseline resistance was determined to be 107/0.33 cm^2^ using a STX2 chopstick electrode connected to a voltmeter. RTgutGC cells (passage number 20-30) were grown in T75 flasks, trypsinized when maximally (>90%) confluent, and cell counts were performed using the trypan blue (0.04%; Thermo Fisher Scientific) exclusion assay and the automated Countess cell counter (Invitrogen) prior to seeding into the cell culture dishes. The cells were seeded on semipermeable Transwell membrane supports (Corning Costar Transwell, Millipore Sigma) at a density of approximately 2.6 x 10^5^ cells/cm^2^ or a final number of about 8.6 x 10^4^ cells per insert (cell growth aera of 0.33 cm^2^). The cells were cultured for at least 3 weeks prior to the experiment to ensure that they established the brush border membrane and tight junction complexes. To the apical and basolateral compartments 100 μL or 500 μL, respectively, of L-15 media supplemented with 10% FBS were added, and the medium was replaced every 4-5 days. Periodic inspection of the cell monolayers was carried out using a Nikon inverted light microscope.

Bacterial cultures were prepared as outlined in section 2.1.2. and bacteria containing cell culture growth medium was added to the RTgutGC cells and incubated for 24 h. At the end of the incubation time, culture medium was carefully removed so not to disturb the cell monolayer and the Transwell inserts were transferred to a new 24-well plate containing sterile PBS on the basolateral compartment. To the apical compartment, 100 μL of PBS were added. Cell monolayers were likewise washed two more times, 100 mL of PBS was added to the apical compartment and 500 μL to the base of the electrode, and measurements were recorded using a cup electrode collected to a voltmeter. The baseline reading (membrane only) was subtracted from the measurements and the resistance per cm^2^ was determined. Statistical analyses were performed on the resistance values per area.

### 2.2 *In vivo P. acidilactici* MA18/5M supplementation trial

#### 2.2.1 Experimental design

**Figure 5:**
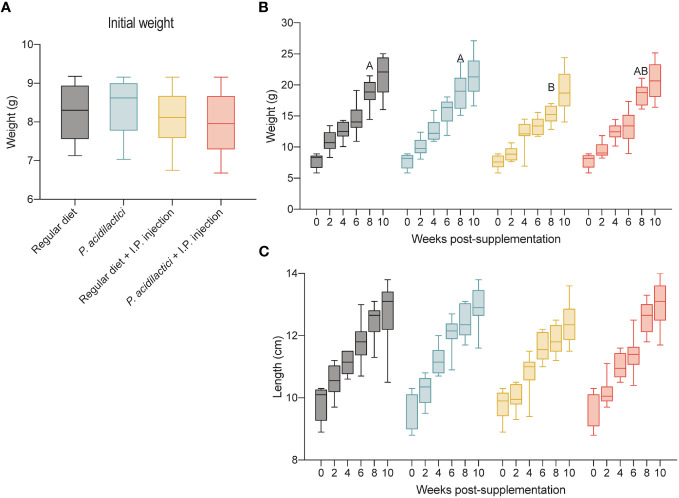
*In vivo* trial in juvenile Chinook salmon with diet supplementation of *P. acidilactici* MA18/5M followed by intraperitoneal challenge with *V. anguillarum* J382. Initial weight (g) of fish transferred to test the different supplementation groups **(A)**. Each column represents an average of 35 fish. Weight (g) increase recorded for a total of 10 weeks post-supplementation **(B)**. Length (cm) increase recorded for a total of 10 weeks post-supplementation **(C)**. All data are expressed as mean values ± S.E.M. Different letters represent significant differences among treatments at the same time (P < 0.05).

Chinook salmon (*O. tshawytscha*) juveniles (n = 280; 8.168 ± 0.721 g) were obtained from Yellow Island Aquaculture Ltd. (www.yellowislandaquaculture.ca, Quadra Island, British Columbia, Canada) and the experiments were conducted there. Before the infection trial, fish were kept in freshwater (FW, 14 ± 2°C) at a density of 2.3 kg/m^3^ using a flow-through system under natural photoperiod (12:12 h dark:light). After this, fish were randomly distributed between eight 120 L barrels (2 barrels per treatment, 35 fish per barrel) one-day prior the starting of feeding trial. During the transfer, the initial fish weight (g) was recorded (**Figure 5A**). To minimize growth differences related to the initial fish size and not to the diet supplementation, only fish around 8 ± 1 g were selected (**Figure 5A**). The experimental diets (treatments) utilized in this experiment were: i) Control diet, ii) *P. acidilactici* (MA18/5M probiotic strain), iii) Control diet intraperitoneally injected with heat-killed *V. anguillarum*, and iv) *P. acidilactici* intraperitoneally injected with heat-killed *V. anguillarum* (Please referrer to **Supplementary Material 1** for formulation details). During the trial, fish were fed commercial dry pellet (EWOS Harmony 2 mm: 47% protein, 18% fat, 0.7% fiber, 2.9% calcium, 1.2% phosphorus, and 0.6% sodium) twice a day with a ration of 2% body weight.

#### 2.2.2 Chinook salmon probiotic and heat-killed *V. anguillarum* stimulation trial

**Figure 6:**
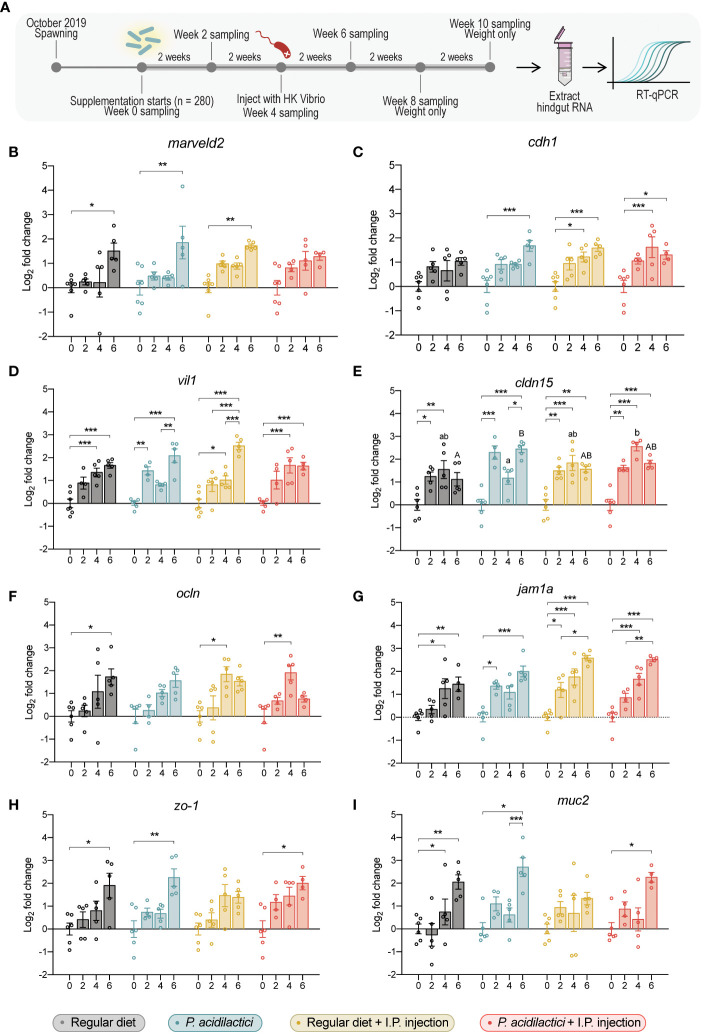
Gene expression analysis of relevant gut barrier genes in the hindgut of juvenile Chinook salmon fed with regular diet or supplemented with *P. acidilactici* MA18/5M and challenged with *V. anguillarum* J382 **(A)**. Transcripts for **(B)** tricellulin (*marveld2*), **(C)** e-cadherin (*cdh1*), **(D)** villin 1 (*vil1*), **(E)** claudin 15 (*cldn15*), **(F)** occludin (*ocln*), **(G)** junctional adhesion molecule 1 alpha (*jam1a*), **(H)** zonula occludens-1 (*zo-1*), and **(I)** mucin 2 (*muc2*) were assessed. All data are expressed as mean values ± S.E.M (n = 6). Bars represent significant differences between time-points at the same treatment. Different letters represent significant differences between treatments at the same time-point (*P < 0.05, ** P < 0.01, *** P < 0.001).

To determine the effect of *P. acidilactici* MA18/5M on Chinook salmon juveniles, both Control diet groups (Control diet and Control diet intraperitoneally injected with heat-killed *V. anguillarum*) and both *P. acidilactici* groups (*P. acidilactici* (MA18/5M probiotic strain) and *P. acidilactici* intraperitoneally injected with heat-killed *V. anguillarum*) were fed with dry pellet or dry pellet supplemented with the probiotic strain respectively for four weeks (**Figure 6A**). After this, to evaluate the effect of probiotic supplementation after inactivated-bacteria stimulation, one control group and one *P. acidilactici* group was injected with heat-killed *V. anguillarum* grown as mentioned in section 2.1.2. The respective diets remained during the total duration of the study.

The timing of sampling for this experiment was 0-, 2-, 4-, and 6-weeks post-diet supplementation (**Figure 6A**). For sampling, fish were exposed to a non-lethal dose of clove oil, and one half of the hindgut tissue was isolated immediately, placed in RNAlater, and stored at -20°C for RNA extraction and qPCR analysis. The other half of the hindgut was placed in 10% neutral buffered formalin (Azer Scientific, Fisher Scientific) at 4°C for further histopathology analysis. Additionally, weight (g) and length (cm) were also recorded at 8-, and 10-weeks post-diet supplementation (**Figure 6A**).

#### 2.2.3 Hindgut RNA extraction and cDNA synthesis

Total RNA was isolated from Chinook salmon hindgut samples using 1 mL of TRIZOL reagent following the manufacturers protocol (Invitrogen, 2020). After extraction, the RNA was treated with Ambion DNase I (RNase free) (Ambion^™^ DNase I, Invitrogen) following the manufacturer’s instructions to degrade any residual genomic DNA. Briefly, 5 μg of RNA was treated with 2 μL of Ambion DNase I, 4 μL of DNase buffer 10x, and DEPC water complete to 40 μL. Then, samples were incubated at 37°C for 30 min, washed twice with Wash solution A, centrifuged at 3,500 x g for 1 min and purified in an RNA/Protein Purification Column. The supernatant containing the RNA was carefully transferred to a new tube. DNase treated RNA samples were quantified and evaluated for purity (A260/280 and A260/230 ratios) using a Take3 plate of a Synergy H1 Hybrid plate Reader (Biotek Instruments, Inc., USA). Column purified RNA samples had A260/280 ratios between 1.9 and 2.1 and A260/230 ratios between 1.9 and 2.2.

First-strand cDNA templates for qPCR were synthesized from 250 ng of DNaseI-treated, column-purified total RNA using qScript cDNA Supermix (Quanta Biosciences) following the manufacturer’s instructions. Each sample was incubated at 25°C for 5 min, at 42°C for 30 min, and at 85°C for 5 min. Samples in a concentration of 25 ng/µL were stored at -20°C until utilization.

#### 2.2.4 qPCR analysis of Chinook salmon hindgut

All qPCR reactions were performed in a 10 μL reaction, 5 µL of 2x WISENT ADVANCED™ qPCR master mix (Wisent, Quebec, Canada), 2.5 µL of forward and reverse primer mix (Sigma Aldrich, USA) at a final concentration of 0.5 µM each, and 2.5 µL of cDNA (2.5 ng/µL, 6.25 ng per reaction). All samples were amplified and detected using the LightCycler^®^ 480 II (Roche, USA). The reaction mixtures were pre-incubated for 2 min at 95°C, followed by 40 cycles of denaturation for 5 s at 95°C, annealing for 30 s at 60°C, and finally extension for 8 s at 72°C. The melt curve was completed for each run every 5 s from 65°C to 97°C.

The primer sequences of interleukin 1 beta (*il1b*), interleukin 6 (*il6*), interleukin 8 (*il8*), interleukin 10 (*il10*), interleukin 17a (*il17a*), tumor necrosis factor alpha (*tnfa*), myeloid differentiation factor 88 (*myd88*), transforming growth factor β (*tgfb*), tricellulin (*marveld2*), E-cadherin (*cdh1*), villin 1 (*vil1*), claudin 3 (*cldn3*), claudin 12 (*cldn12*), claudin 15 (*cldn15*), occludin (*ocln*), junctional adhesion molecule 1 alpha (*jam1a*), zonula occludens-1 (*zo-1*), and mucin 2 (*muc2*) used in this study are listed in **Table 1**. Gene discovery, qPCR primer design, initial quality testing, and primer efficiencies were assessed following the methods mentioned in section 2.1.5.

Transcripts levels of the genes of interest (*il1b*, *il6*, *il8*, *il10*, *il17a*, *tnfa*, *myd88*, *tgfb*, *marveld2*, *cdh1*, *vil1*, *cldn3*, *cldn12*, *cldn15*, *ocln*, *jam1a*, *zo-1*, *and muc2*) were normalized to transcript levels of two endogenous control genes. Levels of five candidate normalizers [elongation factor 1 alpha (*ef1a*), beta actin (*actb*), glyceraldehyde-3-phosphate dehydrogenase (*gapdh*), 18S ribosomal RNA (*18S*), and internal transcribed spacer 2 locus (*its2*)] were assessed in 50% of the samples (i.e., in 3 random samples per treatment) using cDNA representing 6.25 ng of input total RNA. Reference gene stability was assessed using geNorm, NormFinder, BestKeeper, and Delta Ct comparison, through the bioinformatic open-access portal RefFinder (51–55). Most stable genes assessed were *ef1a* and *actb*.

After normalizer testing was completed, transcript levels of the genes of interest were analyzed in the individual study samples, with normalization to both *ef1a* and *actb*. In all cases, levels were assessed (in triplicate) in 7 individual for day 0 and five individuals per treatment per time-point for 2-, 4-, and 6-weeks post-diet time-points using cDNA representing 6.25 ng of input total RNA. On each gene a no RT control was included. Gene expression was determined using the comparative 2^−ΔΔCt^ method (50).

#### 2.2.5 Semi-quantitative and qualitative histological analysis

For Chinook salmon samples collected at 0-, 2-, 4-, and 6-weeks post-diet supplementation, five individuals per treatment were processed further for histology. Each 10% formalin-fixed hindgut sample was cut into 5 equal-sized pieces, dehydrated in an alcohol gradient, cleared in two changes of xylene and sequentially embedded into a single paraffin block. For each fish, 5 μm-thick cross-sections of all five pieces of hindgut were mounted onto a glass slide, stained with hematoxylin and eosin and sealed under a coverslip. All the samples were processed at the Animal Health Laboratory, the University of Guelph (Guelph, ON, Canada).

All analyses were performed using bright field light microscopy on a Leica DMR light microscope (Leica Microsystems Inc., Concord, ON, Canada) equipped with Openlab imaging software (Openlab 5.5, PerkinElmer, Waltham, MA, USA). To visualize the presence of edema, and inflammation of the serosa, submucosa, and lamina propria, samples were observed using a 4x objective (40x magnification). To assess their suitability, the criteria and scales utilized were the following: 0 = none, 1 = mild, 2 = moderate, 3= severe or more extensive than 2. To determine the epithelial vacuolization (vacuolization) and numbers of goblet cells, a 10x objective was used (100x magnification). For epithelial vacuolization, the scores utilized were: 0 = none, 1 = mild vacuolization, 2 = moderate, 3= more extensive than 2 and sometimes involved folding of the epithelium and distortion of the cells and/or folds. For goblet cells, the criteria utilized were: 0 = none visible, 1 = small numbers present as a minority of cells in the epithelium, 2 = greater numbers often grouped together, 3= large numbers or more extensive than 2. Finally, to evaluate the mitotic figures (mitoses), epithelial cell death (apoptosis/necrosis), and multifocal inflammation, a 20x objective was employed (200x magnification). To assess mitoses and apoptosis/necrosis, the numbers were counted for the five intestinal folds.

### 2.3 Statistical analyses

For all RTgutGC *in vitro* trials, data are expressed as mean values ± SEM (n=3). Nonparametric data were statistically compared with a one-way ANOVA (Kruskal-Wallis) and Dunn’s multiple comparisons test. Experiments with two factors were compared with a two-way ANOVA and Tukey’s multiple comparisons test. All statistical analyses were done using GraphPad Prism software v9.0 (GraphPad Software, La Jolla California USA).

For Chinook salmon *in vivo* trials, data are shown as the mean ± SEM (n=5). Assumptions of variance, normality, and homogeneity were tested. A two‐way ANOVA was performed using the different dietary treatments and time-points as factors of variance, followed by a Tukey’s multiple comparisons test to identify differences between groups. All statistical analyses were performed using STATISTICA v7.0 (StatSoft software, Tulsa, USA) and graphs performed using GraphPad Prism v9.0 (GraphPad Software, La Jolla California USA).

Histopathological data were analyzed using a two-way ANOVA using GraphPad Prism v9.0 (GraphPad Software, La Jolla California USA). Different dietary treatments and time-points as factors of variance were considered. A Bonferroni correction was applied, and statistical significance was declared at P ≤ .05 for all dependent variables.

## 3 Results

### 3.1 Effect of coincubation with *P. acidilactici *MA18/5M on the expression of tight junction and immune molecules in RTgutGC cells

To examine whether *P. acidilactici* could promote gut barrier integrity and modulate immune-related genes in the established RTgutGC *in vitro* model, a 48 h endpoint coincubation experiment was performed using differentiated RTgutGC cells (**Figure 1A**) (56). However, *P. acidilactici* did not cause a significant change in the expression of the tight junction genes assessed (*cldn3*, *cldn12*, and *zo-1*) nor did it induce changes in expression of key proinflammatory cytokines (**Figure 1B**).

### 3.2 Changes in transepithelial electrical resistance in response to *P. acidilactici* MA18/5M and *V. anguillarum* coincubation

Differentiated RTgutGC cells cultured on semipermeable Transwell polyester membrane supports (pore size 0.4µm) were exposed to suspensions of the LAB (MOB 1:100; ~4 x 10^6^ CFU/mL) or *V. anguillarum* (MOB 2:1; ~2 x 10^4^ CFU/mL) in L-15 cell culture media for 24h (**Figure 2A**). The *V. anguillarum*, but not *P. acidilactici*, caused an appreciable decrease in resistance relative to the vehicle control (**Figure 2B**).

To determine whether pre-treatment with the candidate probiotic could protect the cell monolayer against the pathogen-induced damages to the intercellular tight junctions, the same strain was grown and added to the apical compartment of the membrane inserts in like manner for 48h. Then, *V. anguillarum* (MOB 2:1; ~2 x 10^4^ CFU/mL) was added for 24 h and TEER measurements were taken at the end of the incubation period (**Figure 2C**). There were no statistically significant differences in resistance in either of the LAB-pretreated group, despite the addition of the pathogen. However, a statistically significant decrease in resistance (P = 0.0265) was observed in the group incubated with *V. anguillarum* only (**Figure 2C**).

### 3.3 Effect of exposure to *V. anguillarum* on the expression of tight junction and immune molecules

To characterize the response of RTgutGC cells to live or heat-killed (HK) *V. anguillarum*, a time-course coincubation experiment was carried out (**Figure 3A**). Samples were collected at 0, 3, 6, and 24 h (**Figure 3A**). Of the TJ-related molecules assessed, the live pathogen elicited a significant downregulation of *cdh1* and *jam1a*, but not *ocln*, which had a puzzling upregulation by the 24 h timepoint (**Figures 3B–D**). Of the cytokines assessed, all exhibited a time-dependent upregulation, which was statistically significant at the 24 h timepoint relative to the 0 h control group (**Figures 3E–G**). In the case of *il8*, but not *il17a* or *tgfb*, the upregulation was observed in cells exposed to both live and HK bacteria (**Figures 3E–G**). In an independent replicate of this experiment, a similar time-dependent response was observed, in which there was a significant upregulation of *il1b*, *il8*, and *tnfa* at the 24 h timepoint for groups exposed to live bacteria (data not shown).

### 3.4 Effect of pre-treatment with LAB and exposure to *V. anguillarum* on the expression of immune and tight junction molecules

To examine the potential use of the LAB as a disease prevention strategy by stimulating immunity and gut barrier function, a time-course coincubation experiment was performed. Briefly, differentiated RTgutGC cells were pre-treated with *P. acidilactici* for 48 h and then exposed to *V. anguillarum*. Samples were taken at 0 h, 3 h, 6 h, and 12 h after infection with the pathogen and the relative expression of key TJ and immune molecules was assessed through RT-qPCR (**Figure 4A**).

The expression of *zo-1* was significantly (p < 0.05) upregulated in the *P. acidilactici* at the 12 h timepoint relative to the expression level at 0, 3, and 6 h (**Figure 4B**). In contrast, a significant upregulation of *cldn3* was observed 6 h post-exposure with *V. anguillarum* compared to 0 and 12 h timepoints (**Figure 4C**). There was a statistically significant (p < 0.05) difference in the expression of *cdh1* for both treatment groups after 12 hpi (hours post-infection) compared to the other timepoints (**Figure 4D**). Lastly, there was also a significant (p < 0.05) downregulation of the *jam1a* molecule 12 hpi in the control and *P. acidilactici* MA18/5M groups compared to 0, 3, and 6 hpi, and 0 hpi respectively (**Figure 4E**). The expression of the pro-inflammatory cytokines *il8* and *il17a* was also assessed. There was an extremely significant (p < 0.05) upregulation of *il8* at the 12 h timepoint in both groups relative to the baseline control and the 3 and 6 hpi timepoints (**Figure 4F**). In contrast, no differences were observed in the expression of *il17a* between treatments and timepoints (**Figure 4G**).

### 3.5 Growth analysis

To minimize growth differences related to the initial fish size and not to the diet supplementation, fish weight (g) was recorded during the transfer to each of the treatments (**Figure 5A**). Overall, no statistically significant differences were observed in weight between the treatments tested in this study (**Figure 5A**).

Results collected during the trial showed significant differences in weight (g) of fish belonging to the regular diet and *P. acidilactici* treatments compared to the regular diet + I.P. injected treatment 8 wps (weeks post-supplementation) (**Figure 5B**). In contrast, no significant differences in weight (g) among treatments were observed at 0-, 2-, 4-, 6-, and 10-wps (**Figure 5B**). Length (cm) data collected during the supplementation trial did not show statistically significant differences between treatments (**Figure 5C**).

### 3.6 Chinook salmon gut-specific relative expression

Transcript levels of gut-specific genes were evaluated by qPCR in hindgut samples (**Figure 6A**). A significant increase in the transcript expression of *marveld2* 6 wps was found in fish from the regular diet, *P. acidilactici*, and regular diet + I.P. injection treatments compared to their respective time 0 (**Figure 6B**). An upregulation on the relative expression of *cdh1* gene was observed at 4 and 6 wps in the regular diet + I.P. injection and *P. acidilactici* + I.P. injection treatments compared to their respective control timepoint (**Figure 6C**), meanwhile, an upregulation of *cdh1* was noted in *P. acidilactici* treated fish at 6 wps compared to time 0 (**Figure 6C**).

The relative expression of the *vil1* encoding gene showed different upregulation patterns among treatments (**Figure 6D**). After 4- and 6- wps, an increase of *vil1* in the regular diet treatment was observed compared to 0 wps (**Figure 6D**). In *P. acidilactici* treatment, *vil1* encoding transcripts in 2 and 6-wps fish showed an upregulation compared to 0 weeks supplemented fish (**Figure 6D**). Moreover, fish supplemented with *P. acidilactici* for 6 weeks showed an upregulation in the *vil1* gene compared to 4 wps (**Figure 6D**). At 4 wps, regular diet + I.P. injection fish showed an increase in *vil1* compared to the time 0 of supplementation (**Figure 6D**). Moreover, 6 wps fish showed an increase in the *vil1* expression compared to 0-, 2-, and 4 wps (**Figure 6D**). Finally, individuals from the *P. acidilactici* + I.P. injection treatment showed an up-regulation after 4- and 6 wps on the expression of the *vil1* gene compared to time 0 (**Figure 6D**).

An increased relative expression of *cldn15* was observed in fish supplemented for 2- and 4 weeks with a regular diet compared to day 0 (**Figure 6E**). In *P. acidilactici* treated fish, the *cldn15* gene showed an upregulation at 2- and 6-wps compared to 0-weeks supplemented fish (**Figure 6E**), whereas, 6-weeks supplemented fish also showed a statistically significant upregulation compared to 4-weeks *P. acidilactici* supplemented fish (**Figure 6E**). For regular diet + I.P. injection and *P. acidilactici* + I.P. injection treatments, a similar pattern of upregulation of the *cldn15* gene was observed at 2-, 4-, and 6-wps compared to their respective time 0 (**Figure 6E**). Interestingly, *cldn15* was the only gene that showed significant differences between diet treatments at the same time point. For instance, a statistically significant upregulation of *cldn15* was observed in *P. acidilactici* + I.P. injection treatment compared to the *P. acidilactici* treatment at 4 wps (**Figure 6E**). Moreover, upregulation of *cldn15* was observed after 6 wps with *P. acidilactici* compared to the regular diet treatment (p < 0.05) (**Figure 6E**).

Transcripts encoding the expression of *ocln* showed statistically significant differences in regular diet treatment between 0 and 6 wps (**Figure 6F**). Also, an upregulation after 4 weeks of supplementation in the relative expression of *ocln* was observed in the regular diet + I.P. injection and *P. acidilactici* + I.P. injection treatments compared to 0 wps (**Figure 6F**). The relative expression of *jam1a* in regular diet treated fish was upregulated after 4 and 6 wps compared to time 0 (**Figure 6G**). In *P. acidilactici* treatment, an upregulation at 2 and 6 wps was found compared to time 0 (**Figure 6G**). For regular diet + I.P. injection treated fish, significant differences were found in the expression of *jam1a* after 2 wps compared to 0 wps (**Figure 6G**). An increased relative expression of *jam1a* was observed in fish under regular diet + I.P. injection or *P. acidilactici* + I.P. injection treatments for 4 and 6 weeks compared to their respectively time 0 (**Figure 6G**). Additionally, an upregulation of this gene after 6 weeks of either regular diet + I.P. injection or *P. acidilactici* + I.P. injection treatments was found (p < 0.05) compared to fish 2 wps (**Figure 6G**).

The relative expression of *zo-1* encoding gene was upregulated after 6 weeks of either regular diet, *P. acidilactici*, or *P. acidilactici* + I.P. injection treatment compared to their correspondingly time 0 (**Figure 6H**). Similarly, an upregulation in the transcripts encoding *muc2* was observed in fish from the regular diet, *P. acidilactici*, and *P. acidilactici* + I.P. injection treatments (**Figure 6I**). In the regular diet treatment, *muc2* relative expression was upregulated after 4 and 6 wps compared to time 0 (**Figure 6I**). For *P. acidilactici*-treated fish, this gene showed a statistically significant increase at 6 wps compared to 0 and 4 wps (**Figure 6I**). Finally, a significant increase in the expression of *muc2* was determined in fish from the *P. acidilactici* + I.P. injection treatment after 6 weeks compared to baseline fish (0 wps; **Figure 6I**).

### 3.7 Chinook salmon gut immune relative expression

**Figure 7:**
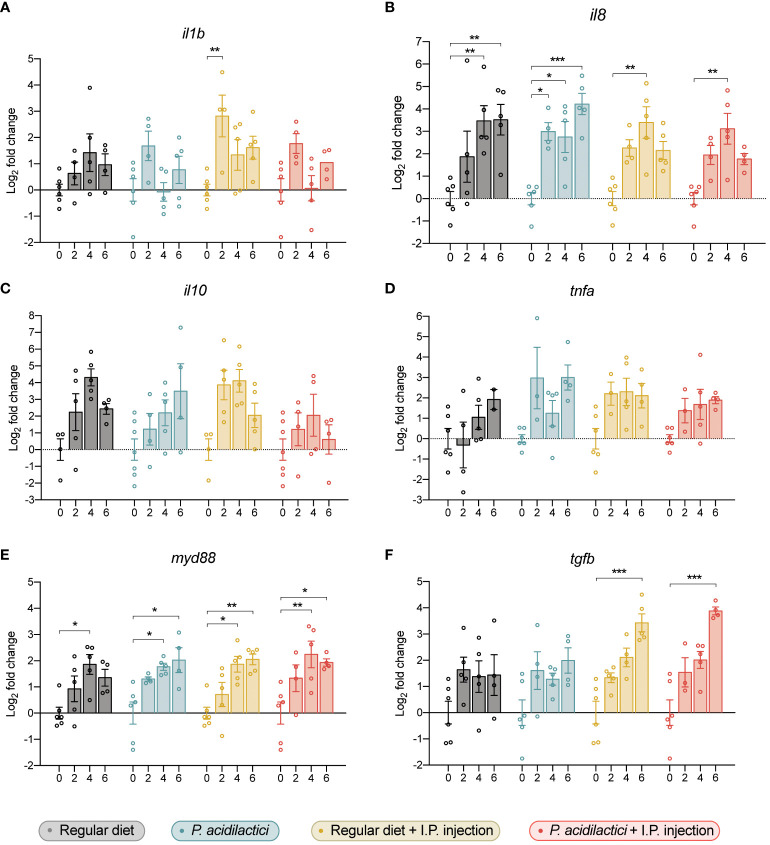
Gene expression analysis of relevant immune genes in the hindgut of juvenile Chinook salmon fed with regular diet or supplemented with *P. acidilactici* MA18/5M and challenged with *V. anguillarum* J382. Transcripts for interleukin **(A)** 1b (*il1b*), **(B)** interleukin 8 (*il8*), **(C)** interleukin 10 (*il10*), **(D)** tumor necrosis factor alpha (*tnfa*), **(E)** myeloid differentiation factor 88 (*myd88*), and **(F)** transforming growth factor β (*tgfb*) were assessed. All data are expressed as mean values ± S.E.M (n = 6). Bars represent significant differences between time-points within treatments (*P < 0.05, ** P < 0.01, *** P < 0.001).

The effect of the *P. acidilactici* MA18/5M strain and of the inactivated pathogen stimulation on Chinook salmon immune genes were evaluated by qPCR (**Figure 7**). In our study, an upregulation of the pro-inflammatory cytokine *il1b* was only observed in the regular diet + I.P. injection treatment at 2 wps compared to time 0 (**Figure 7A**). In contrast, a statistically significant increase in the expression of the pro-inflammatory chemokine *il8* was observed in each treatment (**Figure 7B**). For instance, an upregulation in *il8* was observed after 4- and 6 wps compared to 0-weeks of regular diet treatment (**Figure 7B**). Also, a significant increase in the expression of *il8* was determined after 2-, 4-, and 6 wps with *P. acidilactici* compared to day 0 (**Figure 7B**). In fish sampled from the regular diet + I.P. injection and *P. acidilactici* + I.P. injection treatments, a similar upregulation was observed after 4 wps compared to their respectively time 0 (**Figure 7B**). In contrast, the relative expression of *il10* and *tnfa* was not modulated by the treatments utilized in this study (**Figures 7C, D**).

Transcript levels of *myd88* were upregulated in all conditions after 4 wps compared to time 0 in each treatment (**Figure 7E**). Moreover, an upregulation in the expression of *myd88* compared to time 0 was observed in the *P. acidilactici*, regular diet + I.P. injection, and *P. acidilactici* + I.P. injection treatments (**Figure 7E**). Finally, a statistically significant increase in the expression of *tgfb* was observed in fish sampled from the regular diet + I.P. injection and *P. acidilactici* + I.P. injection treatments at 6 wps compared to time 0 (**Figure 7F**).

### 3.8 Histopathological effects after *P. acidilactici* supplementation and heat-killed pathogen stimulation

**Figure 8:**
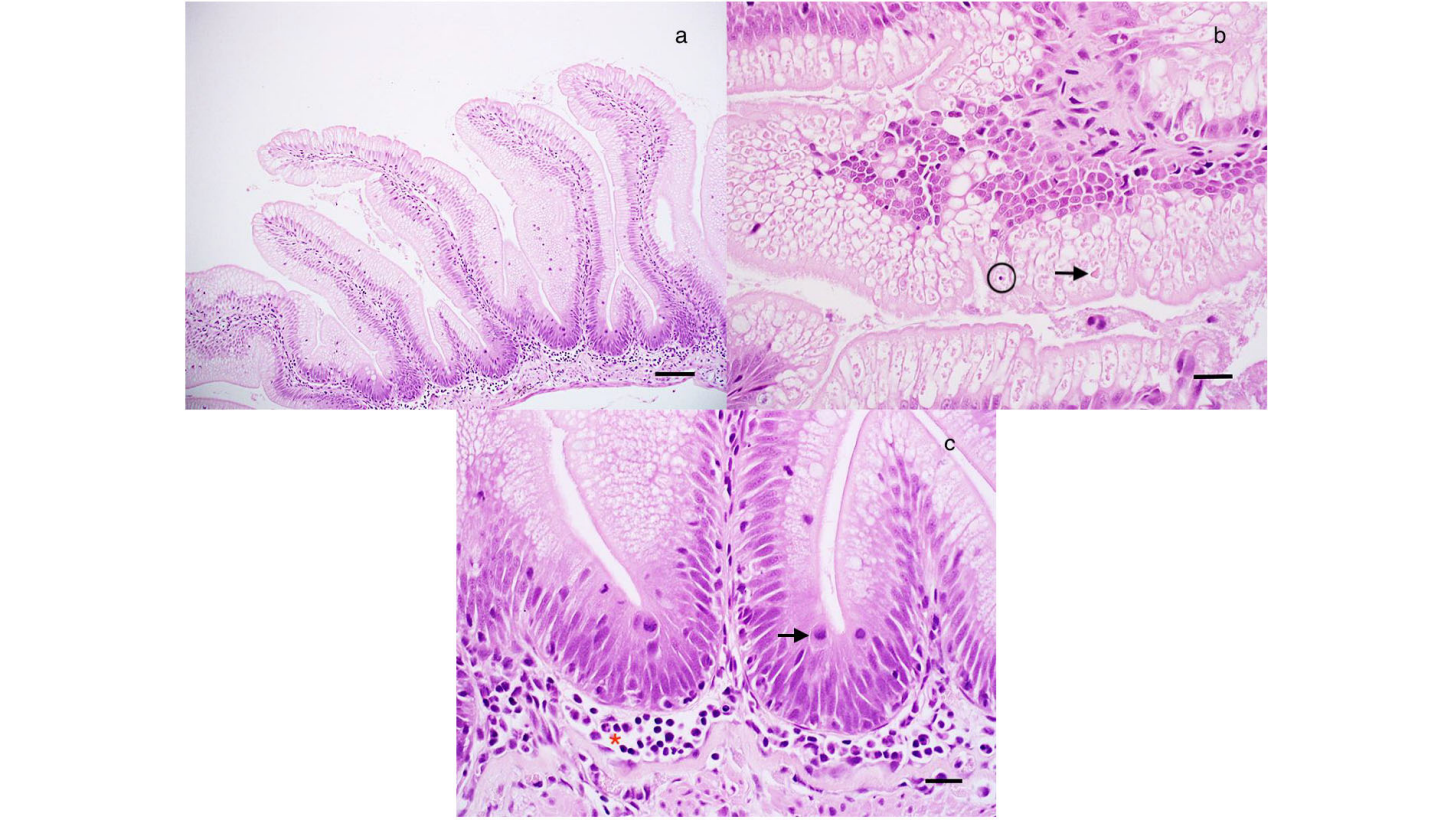
Histological analysis of juvenile Chinook salmon intestine fed with regular diet or supplemented with *P. acidilactici* MA18/5M and challenged with *V. anguillarum* J382. **(A)** Simple intestinal folds with epithelial cells filled with absorptive vacuoles. Bar = 120 mm. **(B)** Atypical intestinal fold with eosinophilic material (arrow) in absorptive vacuoles along with an example of epithelial apoptosis/necrosis, which was uncommon. Bar = 30 mm. **(C)** Detail of a, with numerous mitotic figures in the epithelium (arrow) and a mild increase in inflammatory cells in the submucosa (Red asterisk). Bar = 30 mm.

To examine the effect of *P. acidilactici* supplementation on Chinook salmon hindgut integrity, a histopathological analysis was conducted (**Figure 8**). Moreover, to determine whether *P. acidilactici* could promote hindgut integrity in presence of an immunomodulator, fish from both feeding treatments were I.P. injected with heat-killed *V. anguillarum* (**Figure 8**).

Our results showed that fish feed with the regular diet or *P. acidilactici* that were or not stimulated with heat-killed *V. anguillarum*, did not show evidence of edema, inflammation of the serosa, submucosa and lamina propria, and multifocal inflammation after 0, 4, and 6 weeks (**Table 2**, **Figure 8**). In the case of epithelial vacuolization, although scores associated with moderate (up to half of the cell is filled with absorptive vacuoles) and extensive (involved folding of the epithelium and distortion of the cells and/or folds) vacuolization were reported, no significant differences were observed among treatments and time points (**Table 2**, **Figure 8**). Small numbers of goblet cells were noted in the epithelium and no statistically significant differences were associated to the treatments and time points variables, as well as the interaction of both (**Table 2**, **Figure 8**). In this analysis, the numbers of mitotic figures counted in the five intestinal folds did not show differences among the regular diet, *P. acidilactici*, regular diet + I.P. injection, and *P. acidilactici* + I.P. injection after 0, 4, and 6 weeks of study (**Table 2**, **Figure 8**). There was evidence of epithelial cell death (apoptosis/necrosis) in Chinook salmon hindgut, however, this was not associated with probiotic supplementation and/or stimulation with heat-killed *V. anguillarum* (**Table 2**, **Figure 8**).

**Table 2:** Histopathological analysis of Chinook salmon hindgut (n = 5 per treatment per time-point) during six weeks of dietary and/or stimulation treatment (data is expressed as mean values ± S.E.M).

Time (wps*)		Treatments			
		Regular diet	*P. acidilactici*	Regular diet + I.P injection	*P. acidilactici* + I.P injection
0 weeks	Edema	0.00 ± 0.00	0.00 ± 0.00	0.00 ± 0.00	0.00 ± 0.00
	Inflammation serosa	0.00 ± 0.00	0.50 ± 0.50	0.00 ± 0.00	0.00 ± 0.00
	Inflammation submucosa	0.00 ± 0.00	0.00 ± 0.00	0.00 ± 0.00	0.50 ± 0.50
	Inflammation lamina propria	0.00 ± 0.00	0.00 ± 0.00	0.00 ± 0.00	0.50 ± 0.50
	Vacuolization	3.00 ± 0.00	2.50 ± 0.50	3.00 ± 0.00	2.50 ± 0.50
	Goblet cells	1.00 ± 0.00	1.00 ± 0.00	1.00 ± 0.00	1.00 ± 0.00
	Mitoses	5.55 ± 0.50	4.00 ± 2.00	3.00 ± 0.00	5.55 ± 4.50
	Necrosis/apoptosis	3.00 ± 0.00	4.00 ± 2.00	2.00 ± 0.00	2.00 ± 1.00
	Multifocal inflammation	0.00 ± 0.00	0.00 ± 0.00	0.00 ± 0.00	0.00 ± 0.00
4 weeks	Edema	0.00 ± 0.00	0.20 ± 0.40	0.00 ± 0.00	0.00 ± 0.00
	Inflammation serosa	0.00 ± 0.00	0.00 ± 0.00	0.00 ± 0.00	0.00 ± 0.00
	Inflammation submucosa	0.00 ± 0.00	0.00 ± 0.00	0.00 ± 0.00	0.00 ± 0.00
	Inflammation lamina propria	0.20 ± 0.40	0.00 ± 0.00	0.00 ± 0.00	0.00 ± 0.00
	Vacuolization	2.60 ± 0.80	2.40 ± 0.49	2.60 ± 0.49	2.00 ± 1.27
	Goblet cells	1.40 ± 0.80	1.40 ± 0.49	1.20 ± 0.40	1.80 ± 0.75
	Mitoses	5.60 ± 4.03	3.20 ± 2.04	2.40 ± 3.83	2.60 ± 2.42
	Necrosis/apoptosis	2.00 ± 1.10	2.20 ± 1.94	0.60 ± 0.49	1.60 ± 1.36
	Multifocal inflammation	0.00 ± 0.00	0.00 ± 0.00	0.00 ± 0.00	0.00 ± 0.00
6 weeks	Edema	0.00 ± 0.00	0.00 ± 0.00	0.00 ± 0.00	0.00 ± 0.00
	Inflammation serosa	0.00 ± 0.00	0.00 ± 0.00	0.00 ± 0.00	0.00 ± 0.00
	Inflammation submucosa	0.00 ± 0.00	0.00 ± 0.00	0.00 ± 0.00	0.00 ± 0.00
	Inflammation lamina propria	0.00 ± 0.00	0.00 ± 0.00	0.00 ± 0.00	0.00 ± 0.00
	Vacuolization	3.00 ± 0.00	3.00 ± 0.00	2.40 ± 0.80	3.00 ± 0.00
	Goblet cells	1.00 ± 0.00	1.00 ± 0.00	1.80 ± 0.75	1.00 ± 0.00
	Mitoses	5.33 ± 4.19	6.00 ± 2.83	3.20 ± 2.48	2.00 ± 1.00
	Necrosis/apoptosis	1.33 ± 0.47	1.67 ± 0.94	0.80 ± 0.75	2.00 ± 0.00
	Multifocal inflammation	0.00 ± 0.00	0.00 ± 0.00	0.00 ± 0.00	0.00 ± 0.00

*wps, weeks post-supplementation.

## 4 Discussion

In elucidating the intricacies of salmonid gut dynamics, this study pioneers a distinctive methodology employing transmembrane plates to model the gut environment. Our primary objective was to dissect the impact of *P. acidilactici* MA18/5M on the nuanced aspects of barrier function and integrity within this system. Moving beyond an examination of these fundamental parameters, we probed the expression of tight junctions and immune molecules upon exposure to the LAB. Furthermore, we explore the molecular mechanism through which *V. anguillarum* J382 exerts its disruptive potential on barrier function, unraveling the interplay between pathogens and the gut milieu. We also investigated the protective attributes of *P. acidilactici* MA18/5M against pathogen-induced assaults on the barrier, employing the *in vitro* model RTgutGC. Complementing these *in vitro* analyses, we extend our inquiry into the broader physiological, immunological, and histopathological landscape of juvenile Chinook salmon, broadening our understanding of the intricate relationship between *P. acidilactici* and the salmonid host organism.

Our results suggest that the sole interaction between *P. acidilactici* MA18/5M and RTgutGC cells did not elicit a statistically significant upregulation of immune (*il1b*, *il8*, *il10*, *il6*, *tnfa*, and *tgfb*) and TJ transcripts (*cldn3*, *cldn12*, *zo-1;* **Figure 1**). Moreover, pre-stimulation with *P. acidilactici* MA18/5M dampened the observed upregulation of *il17a* that occurred in presence of *V. anguillarum* J382 (**Figure 4G**), suggesting that *P. acidilactici* MA18/5M is a good candidate to evaluate in Chinook salmon.

Barrier formation and integrity was assessed by TEER measurements, which were in line with levels reported previously (37, 39, 57). Since the expression of barrier-forming TJ molecules was unchanged, an increase in resistance was not expected. A significant decrease in resistance was observed in cells exposed only to *V. anguillarum* J382 for 24 h, but pre-treatment with *P. acidilactici* MA18/5M for 48 h prior to exposure to the pathogen mitigated these effects (**Figure 2**). These results suggest that *P. acidilactici* MA18/5M can protect the epithelial barrier against pathogen-induced insults. The significant decrease in resistance given by *V. anguillarum* J382 is expected since the arsenal of virulence factors expressed by this bacterium are believed to degrade the epithelial barrier to gain access to the circulation (58). Improvements in barrier function have been reportedly associated with increased levels of related tight junction gene (*cldn3* and *cdh1*) and protein (Claudin-3) levels (37, 39, 57). Although the data in the present study are in apparent contrast with these findings, the effect observed is modest and incubation with the probiotic strain protected against pathogen-induced damage but did not increase resistance after a 48-h incubation period. Further investigation of this phenomenon through orthogonal methods (such as Lucifer Yellow dye translocation studies) would be useful to ensure that the changes observed are physiologically relevant.

The effects of the fish pathogen *V. anguillarum* J382 were assessed in RTgutGC cells grown on conventional culture plates. Following a time-course coincubation experiment with live or heat-killed *V. anguillarum* J382, it was found that *e-cadherin* and *jam-1a* were significantly downregulated by 24 h post-infection in the live group relative to the heat-killed group and the baseline control (**Figure 3**). Interestingly, the expression of gut barrier protein *occludin* was extremely significantly increased in the live group at the 24 h timepoint. These data seem to suggest that *V. anguillarum* J382 not only impairs the barrier integrity, but the pathogen can also inhibit the expression of key barrier-forming TJ molecules.

The role of *occludin* in fish is not well understood, but studies in other organisms suggest that this protein is not only an integral component of tight junctions in various tissues, but that it can also participate in tight junction remodeling in response to cytokines (59, 60). High levels of proinflammatory cytokines, such as TNFA and IFNG, promote the endocytosis of OCCLUDIN in the tight junction complexes, which coincides with increases in tight junction permeability (61). Moreover, cytokine-induced changes in TEER and flux are directly proportional to OCCLUDIN levels (60). Paradoxically, the results in the present study appear to be at odds with the observations previously reported, in which the increased expression of *occludin* given by live *V. anguillarum* J382 exposure is associated with a decrease in resistance. Tight junctions have complex regulatory networks that dynamically respond to physiological stimuli (59). Therefore, post-transcriptional and post-translational modifications can impact the biological function of the junctions. Analyses that consider not only the molecular phenomena impacting barrier function, but also the dynamic nature of these intercellular junctions, would be instrumental in understanding how the gut epithelium responds to threats and activates immune defense mechanisms.

There was a robust upregulation of *il17a* and *tgfb* assessed by the 24 h timepoint for cells incubated with live *V. anguillarum* J382. Additionally, there was a time-dependent increase in the expression of *il8* for both live and heat-killed groups, and these levels were significantly higher by 24 h (**Figure 3**). These results seem to indicate that *il17a* and *tgfb* are involved in the response to secreted virulence factors, whereas *il8* might be more involved in the response to cell wall components such as LPS. These results are in line with the proposed mechanism of *il8* induction given by LPS in other organisms (62). The role of *il17a* secreted by intestinal epithelial cells is less clear. In mammals, *il17a* is produced by a subset of T helper cells that induce the production of antimicrobial peptides, among other proinflammatory molecules (63). Host stimulation by LPS, peptidoglycans, and other antigens through pattern recognition receptors enables antigen-presenting cells to activate naïve T cells that mediate the adaptive immune response to the threat (63). Increased expression of *il17a* is also related to increased permeability of the blood brain barrier and small intestinal epithelial barrier (64). In the context of the present study, pathogen-induced upregulation of *il17a* can potentially enhance the damage to the epithelial barrier and thus contribute to the establishment of the infection.

A coculture experiment was employed to simulate a scenario in which LAB supplementation precedes pathogen exposure. Pre-treatment with LAB has been associated with protection from pathogen-induced injury to the intestinal epithelium *in vitro* (65). The data presented here suggest that the LAB strain tested has a mild effect in preventing pathogen-induced changes in the expression of key barrier proteins (**Figure 4**). Slight differences in trends of expression of *zo-1* were observed in cells pre-treated with *P. acidilactici* 18MA/5M relative to the pre-treatment control group (i.e. *Vibrio* only), indicating that this strain can potentially induce the expression of this key tight junction molecule despite the presence of the pathogen. The expression of *il-8* was highly increased by the 12h timepoint, indicating that the LAB strain tested was unable to dampen the excessive immune activation caused by *V. anguillarum* J382 that can lead to epithelial injury and further contribute to the infection.

To determine the effect of *P. acidilactici* MA18/5M on live fish health parameters, juvenile Chinook salmon reared under aquaculture conditions were supplemented for four months to determine the effect of this strain at physiological and immunological level. Fish growth is a key variable for the aquaculture industry, so it was imperative to start the study with similar size fish for every treatment. No differences associated to the candidate probiotic supplementation were seen during the trial. A previous *P. acidilactici* MA18/5M supplementation study performed in Atlantic salmon, showed that a 12-week supplementation with *P. acidilactici* MA18/5M did not improve the growth, specific growth rate (SGR), and thermal growth coefficient (TGC) (20). This, in addition to our findings in Chinook salmon, suggest that a longer supplementation time might be required to positively impact the fish growth desired for production. Confirmation that the strain is present and metabolically active would also be useful to rule out it simply passing through the gut due to rapid transit or too low a temperature for rehydration.

In the past, inflammation associated with the presence of antinutritional factors in plant ingredients used to feed salmonids (e.g. soybean meal products, soy protein concentrate) has increased the focus in evaluating possible inflammatory processes induced by external products in the fish intestine (66–68). Although it is unknown whether enteritis can be induced by probiotic supplementation, a histopathological analysis is of great importance to determine the safety of *P. acidilactici* MA18/5M supplementation in Chinook salmon. When *P. acidilactici* MA18/5M was added to the diet, there were no histological alterations, indicating that probiotic supplementation did not change the gut morphology in comparison to the regular pellet. Overall, in addition to the *in vitro* data indicating that *P. acidilactici* MA18/5M pre-treatment can protect the epithelial barrier against pathogen-induced damage, our study demonstrated that *P. acidilactici* MA18/5M supplementation does not substantially affect the gut epithelial barrier, making it a great candidate for future supplementation in Canadian aquaculture.

The fish pathogen, *V. anguillarum*, is well known for inducing immune transcripts of the inflammatory response when infecting salmonids (26, 69). Therefore, it is not surprising to see an increase in the expression of *il1b*, *il8*, and *myd88* after inoculation with heat-killed *V. anguillarum* J382. Following this, upregulation of *tgfb*, a suppressor of the activation, proliferation, and function of T-cells to protect the organisms from inflammation is expected (70). However, we observed increases in the expression of these genes in non-injected fish. Even though more evidence is needed to confirm the results, we hypothesize that variation in the transcript encoding the genes seen here, is more likely to be associated with an ontogenetic process during the parr/smolt transition, instead of the treatments. A study conducted in coho salmon (*Oncorhynchus kisutch*) proposed that variations in the hepatic gene expression profiles observed in smolts and adults might be associated to complex physiological transformations as the fish start preparing to migrate towards seawater (71). Also, Johansen et al. (72) obtained differential expression of chemokines and antiviral genes in uninfected Atlantic salmon parr and smolts.

Similar to the immune genes evaluated in this study, TJ genes *marveld2*, *cdh1*, *vil1*, *cldn15*, *ocln*, *jam1a*, *zo-1*, and *muc2* did not show a modulation pattern associated with probiotic supplementation, the heat-killed *V. anguillarum* J382 stimulation, or both. As hypothesized above, the parr/smolt transition represents an important physiological change in Chinook salmon, therefore, for genes with a complex regulatory network, such as TJ, variations can be extensive during this process. Since parr/smolt transformation represents a stressful stage that affects the intestinal homoeostasis of salmonids (20, 43), expected effects on gut barrier function after *P. acidilactici* MA18/5M supplementation might be masked by ontogenetic changes. To avoid this, future research should focus in determining the effect of *P. acidilactici* in adult Chinook salmon already transferred to sea pens.

## 5 Conclusion

The *in vitro* coculture system is a powerful and cost-effective tool for the investigation of host-microbe interactions. This study is the first of its kind to employ a tissue culture of the salmonid intestine in a semipermeable membrane system (Transwell) for investigating host-microbe interactions and evaluating the potential of *P. acidilactici* MA18/5M as a fish probiotic. Gene expression of immune and TJ genes and histopathological analysis supported the findings obtained in RTgutGC, showing that *P. acidilactici* MA18/5M supplementation does not negatively impact Chinook salmon homeostasis. Future immunostimulant research should focus in evaluating an increased number of TJ genes in addition to canonical immune response transcripts, since during probiotic supplementation, the gut plays a primary role in the host-microbe interaction.

In the context of this study, the *P. acidilactici* strain examined did not exhibit a strong ability to modulate barrier function in a salmonid intestinal cell line or enhance health parameters in live juvenile Chinook salmon. Importantly, since intraperitoneal infection does not simulate what occurs in natural environments, studies would benefit from using a different delivery method for infection, such as bath immersion, to compare the results obtained by our group. Although no deleterious effects were observed, the benefits provided to fish must be of sufficiently large magnitude to offset the costs in sourcing and administering the beneficial microbes and therefore make probiotic supplementation a viable, sustainable, and affordable solution for the industry.

## Data availability statement

The datasets presented in this study can be found in online repositories. The names of the repository/repositories and accession number(s) can be found in the article/**Supplementary Material**.

## Ethics statement

The animal study was approved by University of Waterloo Animal Care Committee. The study was conducted in accordance with the local legislation and institutional requirements.

## Author contributions

MS-D: Writing – original draft, Methodology, Investigation, Formal analysis, Conceptualization. LL: Writing – original draft, Methodology, Investigation, Formal analysis, Conceptualization. JH: Writing – review & editing, Resources, Methodology, Funding acquisition. JL: Writing – review & editing, Methodology, Investigation. GR: Writing – review & editing, Supervision, Methodology, Funding acquisition, Formal analysis. BD: Writing – review & editing, Supervision, Resources, Project administration, Methodology, Funding acquisition, Conceptualization.
