# Supplementary material for: The effects of Pediococcus acidilactici MA18/5M on growth performance, gut integrity, and immune response using in vitro and in vivo Pacific salmonid models
Source: Front Immunol. 2024 Mar 27;15:1306458. doi: 10.3389/fimmu.2024.1306458 (PMC11006089; doi:10.3389/fimmu.2024.1306458)
Supplement: Supplementary file 1 [file Table_1.docx]

**Supplementary Material 1.** EWOS harmony 2 mm feed

| **Feed ingredient** | **Restriction** | **Amount of total feed** |
| --- | --- | --- |
|  |  |  |
| Crude Protein  Crude Fat  Crude Fibre  Vitamin A  Vitamin D_3_  Vitamin E  Calcium  Phosphorus  Sodium  Selenium | Minimum  Minimum  Maximum  Min. IU/kg  Min. IU/kg  Min. IU/kg  Actual  Actual  Actual | 47%  18%  0.7%  12,000  4,500  450  2.9%  1.2%  0.6%  0.1 mg/kg |

- For Biopower supplementation, 1 g of probiotic was added to 100 g of dry pellet for a final concentration of probiotic 10^8^ CFU/g of feed.
- Manufactured by: EWOS Canada Limited 7721 – 132nd street, Surrey, British Columbia, Canada, V3W 4M8.
